# *SLC6A4* 5HTTLPR Polymorphism Affects Insulin Secretion in Patients with Polycystic Ovary Syndrome

**DOI:** 10.1155/2018/6130487

**Published:** 2018-07-17

**Authors:** Barbara Šenk, Katja Goričar, Nika Aleksandra Kravos, Mojca Jensterle Sever, Andrej Janež, Vita Dolžan

**Affiliations:** ^1^Faculty of Medicine, Institute of Biochemistry, Pharmacogenetics Laboratory, University of Ljubljana, Vrazov Trg 2, 1000 Ljubljana, Slovenia; ^2^Department of Endocrinology, Diabetes and Metabolic Diseases, University Medical Center Ljubljana, Zaloška Cesta 7, 1000 Ljubljana, Slovenia; ^3^Faculty of Medicine, University of Ljubljana, Vrazov Trg 2, 1000 Ljubljana, Slovenia

## Abstract

**Purpose:**

To investigate in a pilot study of genetic polymorphisms in serotonin system influencing basal- and glucose-stimulated insulin secretion in women with polycystic ovary syndrome (PCOS).

**Methods:**

A cross-sectional study included 65 female patients with PCOS followed up at the endocrine outpatient clinic of the University Medical Center Ljubljana and a control group of 94 young healthy female blood donors. Oral glucose tolerance test was performed only in PCOS patients and basal- and glucose-stimulated blood glucose and insulin levels were measured. All the subjects were genotyped for *5HTR1A* rs6295, *5HTR1B* rs13212041, and *SLC6A4* 5HTTLPR polymorphisms in the serotonin system.

**Results:**

Genotype distributions were in accordance with the Hardy-Weinberg equilibrium (HWE), except for *5HTR1A* rs6295 in healthy controls and *5HTR1B* rs13212041 in PCOS patients that were not consistent with HWE. *SLC6A4* 5HTTLPR polymorphism was significantly associated with insulin secretion (*p* = 0.030) and with the area under the curve of insulin blood levels during OGTT (*p* = 0.021). None of the investigated polymorphisms was significantly associated with basal- or glucose-stimulated blood glucose levels at any point in time during OGTT or with the basal insulin concentration.

**Conclusions:**

Serotonin system may play a role in glucose-stimulated insulin secretion in patients with insulin resistance (IR) and decreased insulin sensitivity. Further studies are needed to conclude whether the observed effect is characteristic for PCOS-related metabolic disturbances or for the identified mutation in different high metabolic risk populations.

## 1. Introduction

Polycystic ovary syndrome (PCOS) is a heterogeneous endocrine and metabolic disorder affecting women of reproductive age. It is characterized by hyperandrogenism and chronic anovulation and usually accompanied by metabolic disturbances [1, 2]. The central pathogenetic feature of the syndrome is IR associated with compensatory hyperinsulinemia [3]. Hyperinsulinemia correlates with increased synthesis and biological availability of androgens [1]. PCOS has a strong genetic component. Candidate genes are related to the biosynthesis and metabolism of gonadotropins and sex hormones, obesity, and regulation of energy metabolism, as well as insulin secretion and its action [2].

Animal model and cell culture studies suggest that the serotonin system may play a role in basal- and glucose-stimulated insulin secretion. Mouse model studies suggest that selective serotonin reuptake inhibitors (SSRIs) inhibit insulin secretion and signaling and that they may affect pancreatic *β* cell function and promote apoptosis [4]. Long-term use of SSRIs has been associated with an increased risk of type 2 diabetes (T2D) [5, 6].

Decreased serotonin reuptake or signaling associated with genetic variability of serotonin reuptake transporter (SLC6A4 or 5HTT) or serotonin receptors, respectively, may affect insulin system in a similar way like SSRIs in the serotonin system [4]. However, studies investigating the association between serotonin system polymorphisms and basal- and glucose-stimulated insulin secretion in humans are lacking. The aim of our study was to investigate if *5HTR1A* rs6295, *5HTR1B* rs13212041, and *SLC6A4* 5HTTLPR polymorphisms influence glucose homeostasis and insulin secretion in PCOS patients.

## 2. Subjects and Methods

### 2.1. Study Population

PCOS patients who were treated and followed up at the outpatient clinics of the Department of Endocrinology, Diabetes and Metabolic Diseases at the University Medical Centre Ljubljana were recruited in the study. They had PCOS diagnosed by the European Society of Human Reproduction and Embryology/American Society for Reproductive Medicine criteria (ESHRE/ASRM) [2] and were at least 18 years old, premenopausal, and obese (BMI ≥ 30 kg/m^2^). DNA samples of 94 healthy young female blood donors were used as controls for the genotyping part of the study. The study was approved by the Republic of Slovenia National Medical Ethics Committee and was carried out according to the Helsinki Declaration. All patients were informed of the study aims and provided a written consent before they were enrolled in the study.

### 2.2. Experimental Procedures

A cross-sectional study was performed. Anthropometric data and metabolic and hormonal parameters were obtained at the beginning of the study. An oral glucose tolerance test (OGTT) was performed at the inclusion in the study, and glucose and insulin levels were measured before and after OGTT. Homeostatic model assessment (HOMA_IR_) was calculated, and values greater than 2.0 were considered as indicative of the presence of IR [7].

From most of the patients, a venous blood sample was obtained for DNA extraction at the inclusion in the study together with the blood samples for determination of other biochemical markers. A small number of patients that did not provide the blood sample were asked, retrospectively, to provide buccal mucosa swabs for DNA extraction.

### 2.3. Genotyping

Genomic DNA was extracted from venous blood samples using FlexiGene DNA kit (Qiagen, Hilden, Germany) or from buccal swabs using QIAamp DNA Mini kit (Qiagen, Hilden, Germany). *5HTR1A* rs6295 (-1019C>G) and *5HTR1B* rs13212041 (3′UTR-1997T>C) polymorphisms were genotyped by real-time PCR (KASPar assay, KBioscience, Hoddesdon, Herts, UK) followed by allele discrimination analysis on 7500 Real-Time PCR System AB (Applied Biosystems, Foster City, CA, USA). For the determination of *SLC6A4* 5HTTLPR polymorphism, we amplified the promoter region by PCR on Applied Biosystems 9700 thermal cycler (Applied Biosystems, Foster City, CA, USA) and checked the amplicon lengths using agarose gel electrophoresis to identify the L allele-specific 528 bp fragment and the S allele-specific 484 bp fragment [8].

### 2.4. Statistical Analysis

The distribution of categorical data was presented with the frequencies, while the continuous variables were presented with median and interquartile range (25–75th percentile). Minor allele frequency (MAF) was calculated by counting the alleles and expressed as the share. By calculating *p* value for the Hardy-Weinberg equilibrium (pHWE), we investigated genotype frequency distribution in the population. We used the *χ*2 test or logistic regression to determine the differences in the distribution of categorical data. To determine whether the data were normally distributed, we used the Shapiro-Wilk test. The values *p* < 0.05 indicated that the data were not normally distributed, and in these cases, nonparametric Mann-Whitney *U* test was used for statistical analysis. We used a dominant genetic model for all the analyses, which means that homozygotes for the wild-type allele were compared to heterozygous and homozygous carriers of the polymorphic allele. All statistical analyses were performed using IBM SPSS Statistics version 19.0 (IBM Corporation, Armonk, NY, USA). The differences were considered statistically significant at *p* value < 0.05.

## 3. Results

A total of 87 women with PCOS participated in the study in the beginning. In 80 patients, DNA was obtained from a venous blood sample at the time of inclusion in the study; while in 7 patients, buccal mucosa swabs were obtained for DNA extraction. The final study group consisted of 65 women with PCOS, median age 30.0 (25.8–36.0) years, who had available data on glucose and insulin blood concentrations as well as genotype data. These patients were obese with BMI of 38.2 (33.5–41.6) kg/m^2^, had increased waist circumference of 121.5 (113.0–130.3) cm, and increased VAT area of 155 (122–201) cm^2^. The majority of patients had insulin resistance; the median HOMA_IR_ was 2.67 (1.42–4.30). In addition, 94 healthy blood donors whose median (25%–75%) age was 23.0 (20.8 to 25.0) were also included in the molecular-genetic part of the study, but OGTT test was not performed in this group.

The genotype frequency distribution for the investigated polymorphisms in healthy controls and in patients with PCOS and their minor allele frequencies (MAF) is shown in Table 1. The genotypes' distribution was in HWE, except for the distribution of *5HTR1A* rs6295 genotypes in healthy controls and *5HTR1B* rs13212041 in PCOS patients that were not consistent with HWE.

Basal- or glucose-stimulated blood glucose levels at any point of time during OGTT were not significantly associated with the investigated polymorphisms. Similarly, none of the investigated polymorphisms significantly affected the area under the curve of glucose levels during OGTT (Table 2).

**Figure 1:**
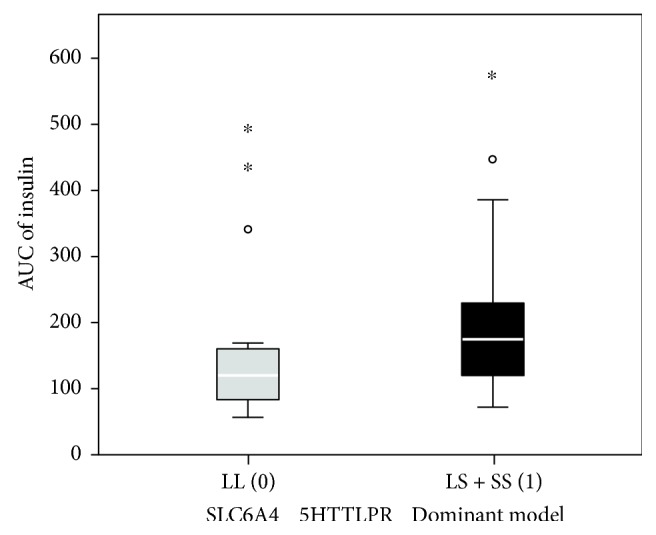
AUC of blood insulin levels during OGTT in relation to 5HTTLPR polymorphism. 0: LL genotype; 1: LS and SS genotypes.

**Figure 2:**
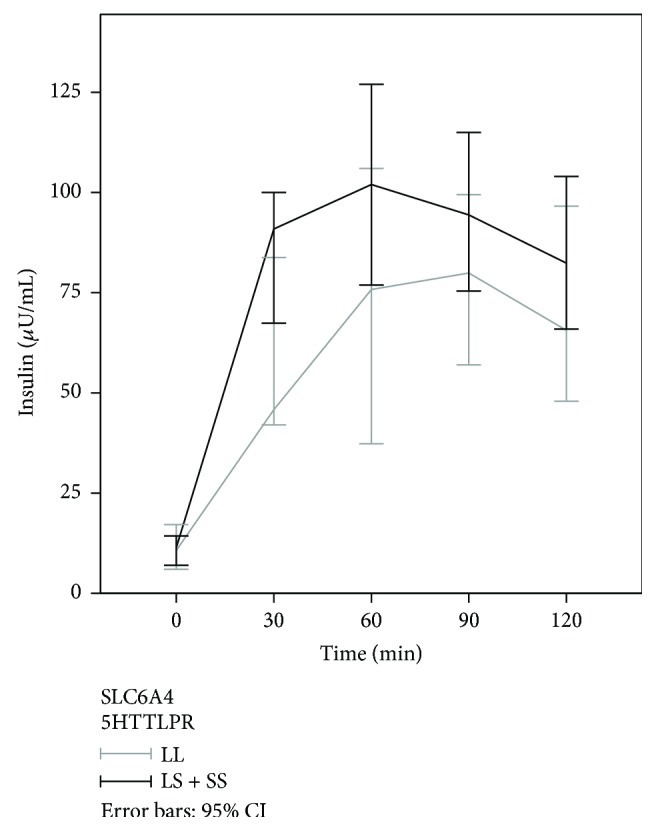
Insulin concentrations during OGTT in relation to 5HTTLPR polymorphism.

None of the investigated polymorphisms was statistically significantly associated with the basal insulin concentrations (Table 3). After glucose stimulation, plasma insulin levels were the highest between the 60th and 90th minute in all patients and then started to decline. *5HTR1A* rs6295 and *5HTR1B* rs13212041 polymorphisms were not associated with glucose-stimulated plasma levels at any time point of the OGTT test. These two polymorphisms had also no effect on the area under the curve of the blood insulin concentration (AUC). 5HTTLPR polymorphism was marginally associated with higher insulin concentration in the 30th minute after the glucose load (*p* = 0.056), while its impact on the insulin concentration in the 60th minute after the glucose load was statistically significant (*p* = 0.030). Higher insulin concentrations were observed in the carriers of at least one polymorphic allele (LS or SS genotype) also in the 90th and 120th minute after the glucose load when compared with the homozygous LL genotype, but the differences were no longer statistically significant (*p* = 0.129 and *p* = 0.400). 5HTTLPR polymorphism was statistically significantly associated also with the insulin AUC (*p* = 0.021) as shown in Table 3 and Figure 1. As shown in Figure 2, in patients with the 5HTTLPR LL, genotype glucose-stimulated insulin levels started to rise later, and the peak levels were lower compared to carriers of at least one polymorphic 5HTTLPR allele (LS and SS genotypes) in which the glucose-stimulated insulin levels started rising faster and more steeply and reached the maximum peak levels already in the 60th minute after the glucose load.

## 4. Discussion

The important novel finding of our study is that *SLC6A4* 5HTTLPR polymorphism influences glucose-stimulated insulin secretion. To our knowledge, this is the first study investigating the selected polymorphisms of the serotonin system in women with PCOS. *SLC6A4* 5HTTLPR polymorphism was significantly associated with insulin secretion during OGTT, whereas none of the investigated polymorphisms was significantly associated with basal- or glucose-stimulated blood glucose levels at any point in time during OGTT or with the basal insulin concentration. The results are indicative for association of genetic polymorphisms in serotonin system with higher functional insulin sensitivity in the dynamic metabolic environments. To date, the influence of the serotonin system on insulin secretion has been shown only in the animal models and cell cultures. Studies in mouse models suggest that SSRIs inhibit secretion, signaling, and action of insulin. They also suggest that SSRIs inhibit the functioning of pancreatic *β* cells and promote their apoptosis. SSRIs also inhibited glucose-stimulated insulin secretion in rodent and human pancreatic islets [4]. It was shown in a rat model that 5HTR receptors can affect the function and proliferation of cells of the pancreas via the sympathetic nervous system [9]. It was also shown that serotonin inhibits insulin secretion after the glucose load in the rat [10]. The presence of the 5HTR1A protein was confirmed in isolated human islets of Langerhans [11]. 5HTR1 selective agonist sumatriptan inhibits the functioning of the exocrine and endocrine pancreas [12]. It was reported that serotonin also affects the functioning of the cells of the immune system, and 5HTR1A protein was found in Jurkat cells [13]. Studies have shown that *5HTR1A* polymorphisms can affect insulin release and T cell activity thereby increasing the risk of developing T1D [14].

In humans, in most studies so far, polymorphisms in the serotonin system have been examined mostly as genetic factors that determine the response to treatment and the severity and extent of adverse events in patients taking antipsychotics or antidepressants [4, 15–18]. Metabolic syndrome with IR is a common adverse event associated with SSRI treatment which indicates a link between the serotonin and insulin system [5, 6]. On the other hand, evidence suggests a bidirectional relationship between IR and depression risk in PCOS patients which also indicates the association between serotonin and insulin system [19]. Our data may also suggest that pharmacological modulation of serotonin system could be used to modulate insulin secretion or reduce IR in women with PCOS in particular when depressive symptomatology is accompanied that is common in these patients and presumably also in other conditions with metabolic disorders associated with IR and depressive symptomatology at the same time. Further studies are needed to determine, if according to the genotype of women with PCOS, substances acting on the serotonin system could be used instead of or as an add-on to antidiabetic therapy to improve insulin resistance, particularly when in addition to metabolic disorders depressive symptomatology is present.

Genetic variability in serotonin system may also have other implications for patients with PCOS of which many decide for in vitro fertilization due to fertility problems. It was reported that among the recipients who underwent in vitro fertilization with donated oocytes, carriers of 5HTTLPR LL genotype, *5HTR1A* rs6295 CC genotype, or two loci LC haplotype had lower achievement and maintenance of pregnancy [20]. Thus, the information on both polymorphisms could help to identify a group of PCOS patients who may have more difficulties with conception and maintenance of pregnancy.

The major limitation of our study was a relatively small number of patients; therefore, our observations need to be validated in a much larger sample. Another limitation is that OGTT test was not performed in healthy controls, so we cannot conclude whether the observed effect is characteristic only for patients with IR or would also be observed in healthy population. Future studies should thus include also healthy controls and both men and women. Despite these limitations, the group of patients was clinically well defined. All patients were monitored in the same institution and by the same endocrinologist, so there were no differences in the clinical assessments. Also, all the analyses were carried out in the same laboratory, so there were no deviations of laboratory parameters due to potential differences in analytical procedures. Furthermore, all the patients and controls came from an ethnically homogeneous Slovenian population, so there was no bias due to genetic heterogeneity [21].

In conclusion, in our pilot study, we have observed that *SLC6A4* 5HTTLPR polymorphism influences insulin secretion in PCOS patients, indicating that serotonin system may play a role in insulin secretion in humans. Further studies are needed to elucidate more clearly how genetic variability within serotonin system and pharmacological interventions with SSRIs modulate glucose-stimulated insulin secretion.

## Figures and Tables

**Table 1:** Genotype frequencies in healthy controls and PCOS patients.

Polymorphism	Healthy controls				PCOS patients			
	Genotype	Number (%)	MAF	pHWE	Genotype	Number (%)	MAF	pHWE
*5HTR1A* rs6295^a^	CC	13 (14.6)	0.54	**0.020**	CC	21 (33.3)	0.47	0.107
	CG	55 (61.8)			CG	25 (39.7)		
	GG	21 (23.6)			GG	17 (27.0)		

*5HTR1B* rs13212041^b^	TT	51 (57.3)	0.24	0.574	TT	39 (63.9)	0.25	**0.001**
	TC	34 (38.2)			TC	13 (21.3)		
	CC	4 (4.5)			CC	9 (14.8)		

5HTTLPR^c^	LL	22 (26.2)	0.45	0.160	LL	19 (34.5)	0.37	0.128
	LS	48 (57.1)			LS	31 (56.4)		
	SS	14 (16.7)			SS	5 (9.1)		

^a^Data for 5 healthy controls and 2 patients are missing. ^b^Data for 5 healthy controls and 4 patients are missing. ^c^Data for 10 healthy controls and 10 patients are missing. MAF: minor allele frequency.

**Table 2:** Serotonin system polymorphisms and blood glucose levels during OGTT.

Polymorphism	Genotype	Fasting blood glucose (mmol/L)		Glucose 30 min (mmol/L)		Glucose 60 min (mmol/L)		Glucose 90 min (mmol/L)		Glucose 120 min (mmol/L)		AUC	
		Median (25%–75%)	*p*	Median (25%–75%)	*p*	Median (25%–75%)	*p*	Median (25%–75%)	*p*	Median (25%–75%)	*p*	Median (25%–75%)	*p*
*5HTR1A* rs6295	CC	5.25 (4.83–5.68)	0.530	9.3 (7.73–10.25)	0.328	9.4 (7.68–10.63)	0.287	8.85 (7.35–9.68)	0.192	7.7 (5.7–8.68)	0.277	16.76 (15.17–18.48)	0.182
	CG + GG	5.1 (4.7–5.5)		8.4 (7.45–9.6)		8.65 (7.08 11.1)		7.9 (6.55–9.33)		6.9 (5.8–7.93)		15.66 (13.64–18.09)	
*5HTR1B* rs13212041	TT	5.2 (4.8–5.6)	0.456	8,8 (7.45–9.6)	0.647	9 (7.35–10.23)	0.568	8.55 (6.68–9.53)	0.518	7.35 (6.35–8.53)	0.105	16.39 (13.98–17.96)	0.485
	TC + CC	5.05 (4.6–5.5)		9 (7.35–10.23)		9 (7.55–10.03)		7.75 (6.53–9.53)		6.95 (5.4–7.48)		15.36 (13.63–18.61)	
5-HTTLPR	LL	5.3 (4.9–5.7)	0.334	8.9 (6.9–10.4)	0.601	9 (7.2–10.1)	0.797	8.8 (7.1–9.5)	0.608	7.4 (7–8.2)	0.495	16.65 (14.03–17.58)	0.972
	LS + SS	5.1 (4.7–5.6)		8.9 (7.9–10.2)		9 (7.4–11.1)		8 (6.7–9.5)		6.8 (5.8–8.6)		16.08 (13.78–18.5)	

**Table 3:** Serotonin system polymorphisms and blood insulin levels during OGTT.

Polymorphism		Fasting blood insulin (*μ*U/mL)		Blood insulin 30 min (*μ*U/mL)		Blood insulin 60 min (*μ*U/mL)		Blood insulin 90 min (*μ*U/mL)		Blood insulin 120 min (*μ*U/mL)		AUC	
		Median (25%–75%)	*p*	Median (25%–75%)	*p*	Median (25%–75%)	*p*	Median (25%–75%)	*p*	Median (25%–75%)	*p*	Median (25%–75%)	*p*
*5HTR1A* rs6295	CC	10.35 (7.46–19.48)	0.729	64.75 (41.43–104.75)	0.786	87.95 (52.13–128)	0.461	90.55 (72.1–113.5)	0.886	79.65 (46.83–95.88)	0.547	159.76 (93.35–226.61)	0.740
	CG + GG	11.8 (6.37–19.03)		74.05 (43.05–102.5)		98.5 (66.98–134)		80.75 (61.13–118.5)		72.6 (51.4–125.25)		144.26 (118.27–210.94)	
*5HTR1B* rs13212041	TT	10.95 (7.32–17.95)	0.623	72.3 (4.38–99.25)	0.969	83.95 (59.98–123.25)	0.382	80.55 (61.48–110.5)	0.485	79.3 (55.6–114)	0.539	144.26 (110.79–198.46)	0.602
	TC + CC	11.15 (5.02–20.1)		69.25 (41.6–128.75)		96.45 (60.78–208.5)		91.85 (58.83–180.5)		65.9 (46.38–109.5)		149.63 (108.86–290.86)	
5HTTLPR	LL	10.7 (5.98–17.1)	0.657	45.8 (42–83.8)	**0.056**	75.8 (37.3–106)	**0.030**	79.9 (57–99.5)	0.192	65.6 (47.9–96.6)	0.400	121.53 (78.64–169.2)	**0.021**
	LS + SS	11.4 (6.44–20)		90.9 (54.1–120)		102 (71–137)		94.4 (69.7–120)		82.4 (56–125)		174.98 (120.7–239.25)	

## Data Availability

The underlaying data related to the submission are available by writing to the corresponding authors.
